# Risk factors for intracerebral haemorrhage – Results from a prospective population-based study

**DOI:** 10.1177/2396987320932069

**Published:** 2020-06-12

**Authors:** Edith H Svensson, Kasim Abul-Kasim, Gunnar Engström, Martin Söderholm

**Affiliations:** 1 Cardiovascular Research – Epidemiology, Department of Clinical Sciences, Lund University, Malmö, Sweden; 2 Radiology Diagnostics, Department of Translational Medicine, Lund University, Malmö, Sweden; 3 Department of Radiology, Skåne University Hospital, Malmö, Sweden; 4 Department of Neurology, Skåne University Hospital, Lund and Malmö, Sweden

**Keywords:** Intracerebral haemorrhage, intracranial haemorrhage, stroke, epidemiology, risk factors

## Abstract

**Introduction:**

While the relationship between hypertension and incident intracerebral haemorrhage is well established, other risk factors are less clear. This study examined risk factors for primary intracerebral haemorrhage, separately for lobar and non-lobar intracerebral haemorrhage.

**Patients and methods:**

Incidence of intracerebral haemorrhage was studied among 28,416 individuals from the population-based Malmö Diet and Cancer cohort. Intracerebral haemorrhage cases were ascertained using the Swedish Hospital Discharge Register and the Stroke Register of Malmö, validated by review of hospital records and images, and classified by location by a neuroradiologist. Multivariable Cox regression was used.

**Results:**

Three hundred and thirty-three intracerebral haemorrhages occurred, mean follow-up time was 18.4 years. Systolic blood pressure (hazard ratio per 10 mmHg 1.19 [95% confidence interval 1.13–1.26], diastolic blood pressure (hazard ratio 1.42 [1.27–1.59]), oral anticoagulants (hazard ratio 4.26 [2.17–8.38]), smoking (hazard ratio 1.45 [1.14–1.87]), living alone (hazard ratio 1.32 [1.04–1.69]) and low apolipoprotein B (hazard ratio per 10 mg/dL: 0.94 [0.90–0.99]) were significantly associated with incident intracerebral haemorrhage after multivariable adjustment. Systolic blood pressure, smoking and oral anticoagulants were associated with lobar intracerebral haemorrhage. Systolic blood pressure, diastolic blood pressure, living alone and diabetes were associated with non-lobar intracerebral haemorrhage. Diabetes and diastolic blood pressure showed significantly different relationships with lobar and non-lobar intracerebral haemorrhage. Alcohol, apolipoprotein A1, body mass index, waist circumference, physical activity and education were not independently associated with intracerebral haemorrhage.

**Discussion and conclusions:** Blood pressure, smoking, low apolipoprotein B, oral anticoagulants and living alone were associated with intracerebral haemorrhage. Diabetes was associated with non-lobar intracerebral haemorrhage only. Further research is required on differences between lobar and non-lobar intracerebral haemorrhage.

## Introduction

Intracerebral haemorrhage (ICH), which accounts for 10–15% of all strokes, is associated with high mortality and a considerable risk of severe persistent disability.^1^ As available treatments for ICH have limited effect on outcome, knowledge of ICH risk factors is crucial to reduce disease burden.

Hypertension is associated with increased risk of ICH in many studies.^2,3^ However, the associations with incident ICH are less clear for other common cardiovascular risk factors, such as smoking,^4^ diabetes,^5,6^ high alcohol intake,^7,8^ obesity,^3,7^ psychosocial stress,^3,9^ physical activity^3,10^ and lipid levels,^11,12^ and results vary between studies. Risk factors could also differ for lobar and non-lobar ICH.^2,9,13^ Only a few previous prospective cohort studies have analysed risk factors for lobar and non-lobar ICH separately.^2,9,14–17^ The aim of this study was to investigate the relationships between potential risk factors and incident ICH in a prospective cohort. We also separately studied risk factors for ICH with lobar and non-lobar location.

## Patients and methods

The data that support the findings of this study are available after application to the Malmö Diet and Cancer study (MDCS) steering committee at Lund University, Sweden.

### Study population and baseline examination

The MDCS is a population-based cohort.^2^ Between 1991 and 1996, all men aged 46–73 years and all women aged 45–73 years residing in the city of Malmö were invited by mail or newspaper advertisement. Out of an eligible population of 68,095, 30,447 participated. 28,449 completed the baseline examination. We excluded individuals with history of ICH (*n* = 33), leaving 28,416 subjects.

In the baseline examination, blood pressure was measured in the supine position after 10 min of rest, using a mercury-column sphygmomanometer. Waist circumference was measured and body mass index (BMI) was calculated from weight and height. A self-administered questionnaire was used to assess educational level, physical activity and whether the subjects lived alone, as well as use of cigarettes, alcohol and medications. Participants who had self-reported diabetes, reported use of anti-diabetic drugs, or had been diagnosed with diabetes according to national or local registers of diabetes patients, were classified as diabetic.^18^ A physical activity score was calculated.^19^ Individuals in the lowest quartile were considered to have low physical activity. We categorised educational level as primary (≤9 school years), secondary (10–12 years) or university level (>12 years). Participants that smoked regularly or occasionally were classified as current smokers. High alcohol intake was defined as >40 g/day for men and >30 g/day for women. As the distribution of waist circumference differs substantially between men and women, waist was divided into quartiles with sex-specific cut points. Blood samples were taken at the baseline examination and plasma was separated within 1 h and frozen at −80°C. Apolipoproteins A1 and B (apoA1, apoB) were measured using an immunonephelometric assay, with an inter-assay variability of <4.0% for both proteins.

### Incidence of ICH

All individuals were followed up from the baseline examination until first ICH event, death, emigration or end of follow-up (31 December 2014). Cases of ICH during follow-up (incident ICH) were identified by linkage with the Swedish Hospital Discharge Register (International Classification of Diseases 9th edition code 431 and 10th edition I61.0–9) and the local stroke register of Malmö.^2^ The ICH diagnoses were validated by review of hospital records, including images, and were confirmed when computed tomography, magnetic resonance imaging or autopsy showed parenchymal brain haemorrhage. The present study only included primary ICH cases, in which secondary causes (here defined as trauma, tumour, arterio-venous malformation, haemorrhagic infarction, intravenous thrombolysis) were not present based on available workup. Cases of ICH were classified by a senior neuroradiologist according to bleeding location as lobar (cortical or subcortical white matter) or non-lobar (basal ganglia, periventricular white matter, internal capsule, cerebellum or brainstem). Ten ICH cases that occurred outside of Malmö could not be validated in medical records but were still included in the analyses. Fatal cases of ICH outside of hospital were identified in the Causes of Death Register and diagnosed after autopsy. The Swedish Hospital Discharge Register and the Causes of Death Register covered all hospitalisations and deaths in Sweden during the years of follow-up.

### Statistical analysis

Hazard ratios (HRs) were estimated using Cox’s proportional hazards regression, with age as the underlying time scale. All models adjusted for baseline age and sex. The main multivariable-adjusted model also included systolic blood pressure, blood pressure-lowering drugs, current smoking, high alcohol intake, oral anticoagulants, diabetes mellitus, BMI and living alone. Analyses of apoA1 and apoB were also adjusted for lipid-lowering drugs. In analyses of diastolic blood pressure and waist circumference, these variables substituted systolic blood pressure and BMI, respectively. Potential collinearity in the Cox models was examined; tolerance was above 0.7 for all covariates in the multivariable models and no collinearity problem was observed. Risk factors for lobar and non-lobar ICH were analysed in subgroup analyses. To evaluate whether the associations significantly differed between lobar and non-lobar ICH, a modified version of Lunn–McNeil’s method for Cox regression, with multivariable adjustment, was used. This method has been described in detail elsewhere.^20,21^

*P* values <0.05 were considered statistically significant. Stata version 12.0 (StataCorp. 2011. Stata Statistical Software: Release 12. College Station, TX: StataCorp LP.) was used for statistical analyses.

## Results

Three hundred and thirty-three cases of ICH occurred during a mean follow-up time of 18.4 years. The crude incidence rate was 63.7 (95% CI 57.2–70.9) cases/100,000 person-years. Baseline characteristics in all study subjects and in ICH cases are displayed in Table 1. In the entire cohort, mean age ± SD at screening was 58 ±8 years, 60% were women and 62% had hypertension. In ICH cases, mean age at the ICH event was 74 ±8 years (range, 47–91 years), 53% were women and 80% had hypertension at baseline. Haemorrhage location was defined for 268 cases: 117 were lobar and 151 non-lobar.

**Table 1.** Baseline characteristics in study subjects.

	All subjects	ICH	Lobar ICH	Non-lobar ICH
*n*	28,416	333	117	151
Age, years	58.2 (7.6)	61.8 (7.3)	62.3 (7.5)	61.2 (7.4)
Men, *n* (%)	11,224 (39.5)	158 (47.5)	52 (44.4)	77 (51.0)
Systolic BP, mmHg	141 (20)	151 (21)	149 (21)	152 (21)
Diastolic BP, mmHg	86 (10)	90 (11)	87 (11)	91 (11)
BP-lowering drugs, *n* (%)	5,054 (17.8)	75 (22.5)	26 (22.2)	39 (25.8)
Hypertension, *n* (%)	17,483 (62)	267 (80)	89 (76)	125 (83)
Diabetes mellitus, *n* (%)	1,185 (4.2)	19 (5.7)	3 (2.6)	13 (8.6)
Current smoking, *n* (%)	7,946 (28.3)	96 (29.2)	36 (30.8)	39 (25.8)
High alcohol intake, *n* (%)	1,208 (4.3)	19 (5.8)	7 (6.0)	8 (5.3)
Educational level, *n* (%)				
Primary (≤9 years)	19,109 (68.2)	229 (69.6)	85 (73.5)	105 (70.2)
Secondary (10–12 years)	4,925 (17.6)	57 (17.3)	23 (19.7)	26 (17.2)
University (>12 years)	3,978 (14.2)	41 (12.5)	9 (7.7)	20 (13.3)
Living alone, *n* (%)	6,853 (24.7)	100 (30.6)	28 (23.9)	46 (30.7)
BMI, kg/m^2^	25.8 (4.0)	26.1 (4.3)	25.7 (4.3)	26.2 (4.5)
Waist circumference, men, cm	93.8 (12.5)	92.2 (10.8)	92.2 (10.8)	93.5 (10.1)
Waist circumference, women, cm	77.9 (12.1)	79.3 (10.9)	78.8 (10.5)	79.4 (12.0)
Low physical activity, *n* (%)	6887 (25)	82 (25)	34 (29)	34 (23)
Apolipoprotein A1, mg/dL	156.7 (28.2)	153.8 (27.5)	154.7 (24.9)	152.5 (29.5)
Apolipoprotein B, mg/dL	107.2 (26.1)	107.2 (25.3)	106.7 (25.2)	108.6 (26.5)
Lipid-lowering drugs, *n* (%)	873 (3.1)	8 (2.4)	4 (3.4)	3 (2.0)
Oral anticoagulants, *n* (%)	201 (0.7)	9 (2.7)	6 (5.1)	2 (1.3)

ICH: intracerebral hemorrhage; BP: blood pressure; BMI: body mass index.

Numbers are mean (standard deviation) unless otherwise stated.

### Risk factors for ICH

Of the 28,416 individuals included in this study, 27,666 subjects (326 cases) had complete information on all variables in the main multivariable model.

Results from the age- and sex-, and the multivariable-adjusted analyses, respectively, are presented in Table 2. Systolic and diastolic blood pressure, current smoking, oral anticoagulants, living alone and low levels of apoB were associated with increased risk of ICH after multivariable adjustment (Table 2). High alcohol intake was associated with ICH adjusting for age and sex, but became non-significant after adjustment for systolic blood pressure and smoking. ApoA1, BMI, waist circumference, physical activity and educational level were not significantly associated with ICH.

**Table 2.** Hazard ratios for associations with ICH.

	Age- and sex-adjustedHR (95% CI)	Multivariable adjusted^a^HR (95% CI)
Systolic BP, per 10 mmHg	1.19 (1.13–1.26)	1.19 (1.13–1.26)
Diastolic BP, per 10 mmHg	1.41 (1.26–1.57)	1.42 (1.27–1.59)
Hypertension	2.07 (1.58–2.73)	2.04 (1.53–2.72)
Diabetes mellitus	1.37 (0.86–2.19)	1.41 (0.89–2.27)
Current smoking	1.41 (1.11–1.80)	1.45 (1.14–1.87)
High alcohol intake	1.61 (1.01–2.58)	1.49 (0.93–2.38)
Educational level^b^		
Secondary	1.07 (0.80–1.44)	1.10 (0.82–1.48)
University	1.09 (0.78–1.53)	1.20 (0.85–1.68)
Living alone	1.36 (1.07–1.73)	1.32 (1.04–1.69)
BMI^c^		
<18.5 kg/m^2^	0.66 (0.16–2.65)	0.66 (0.16–2.66)
25–30 kg/m^2^	0.93 (0.73–1.18)	0.92 (0.72–1.18)
>30 kg/m^2^	1.14 (0.83–1.57)	1.03 (0.75–1.44)
Waist circumference^d^	1.08 (0.79–1.48)	0.96 (0.70–1.32)
Low physical activity	1.10 (0.86–1.41)	1.07 (0.83–1.37)
Apolipoprotein A1 (per 10 mg/dL increase)	0.96 (0.92–1.01)	0.96 (0.92–1.01)
Apolipoprotein B (per 10 mg/dL increase)	0.96 (0.92–1.01)	0.94 (0.90–0.99)
Oral anticoagulants	3.81 (1.96–7.42)	4.26 (2.17–8.38)

ICH: intracerebral haemorrhage; HR: hazard ratio; CI: confidence interval; BP: blood pressure; BMI: body mass index.

^a^Main model adjusts for age, sex, systolic BP, use of BP-lowering drugs, current smoking, high alcohol intake, use of oral anticoagulants, diabetes mellitus, BMI and living alone. ApoA1 and apoB also adjusted for lipid-lowering drugs. Diastolic BP not adjusted for systolic BP. Waist circumference not adjusted for BMI.

^b^Secondary (10–12 years) and university (>12 years) education compared to primary education (≤9 years).

^c^Compared to reference group (18.5–25 kg/m^2^).

^d^Fourth compared to first quartile.

### Risk factors for lobar and non-lobar ICH

**Figure 1.**
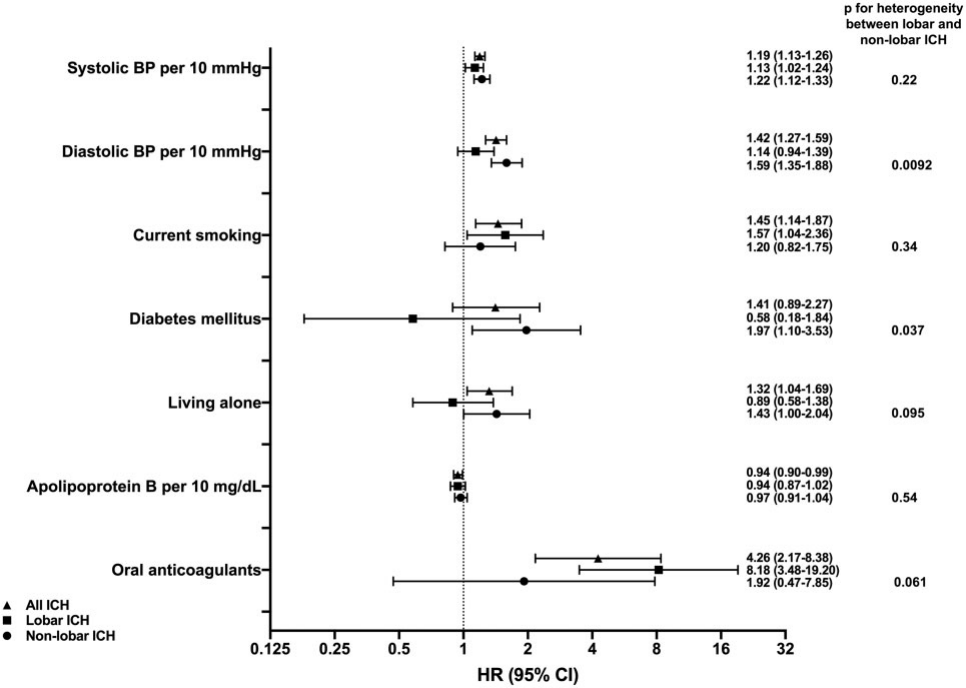
Risk factors significantly associated with all ICH (triangles), lobar ICH (squares) and/or non-lobar ICH (circles) after multivariable adjustment. P-values for heterogeneity of associations with lobar and non-lobar ICH were calculated using a modified version of Lunn–McNeil’s method for Cox regression. Main model adjusts for age, sex, systolic blood pressure, use of blood pressure-lowering drugs, current smoking, high alcohol intake, use of oral anticoagulants, diabetes mellitus, BMI and living alone. Apolipoprotein B also adjusted for lipid-lowering drugs. Diastolic blood pressure not adjusted for systolic blood pressure.

In the multivariable-adjusted analyses, the following variables were significantly associated with lobar ICH: systolic blood pressure (HR per 10 mmHg: 1.13 [confidence interval [CI] 1.02–1.24]), current smoking (HR 1.57 [CI 1.04–2.36]) and oral anticoagulants (HR 8.18 [CI 3.48–19.20]) (Figure 1).

Systolic and diastolic blood pressure (HR per 10 mmHg increase 1.22 [CI 1.12–1.33], and 1.59 [1.35–1.88], respectively), living alone (HR 1.43 [CI 1.002–2.04] and diabetes mellitus (HR 1.97 [CI 1.10–3.53]) were significantly associated with non-lobar ICH (Figure 1). Complete results from the subgroup analyses are presented in Supplement Table 1.

Diabetes (*p* for differential association = 0.037) and diastolic blood pressure (*p* for differential association = 0.0092) had significantly stronger associations with non-lobar ICH than with lobar ICH after multivariable adjustment. For use of oral anticoagulants, living alone, hypertension and systolic blood pressure, *p* values were 0.060, 0.095, 0.15 and 0.22, respectively. For all other risk factors, *p* values were >0.3 for test of difference in effects between lobar and non-lobar ICH.

## Discussion

This prospective population-based study explored risk factors for incident ICH, with subgroup analyses of lobar and non-lobar ICH. High systolic and diastolic blood pressure, current smoking, oral anticoagulants, living alone and low levels of apoB were associated with ICH after multivariable adjustment. The risk factors differed somewhat for lobar and non-lobar ICH; diabetes, diastolic blood pressure and living alone were associated only with non-lobar ICH, while smoking was associated only with lobar ICH. A formal comparison of the differential relationships between ICH of different locations showed that the associations of diabetes and diastolic blood pressure significantly differed between lobar and non-lobar ICH.

ApoB levels were inversely associated with ICH in the multivariable-adjusted analysis, in line with previous studies of LDL or total cholesterol and risk of ICH.^11,13,22,23^ ApoB/apoA1 ratio was associated with only ischemic stroke in the INTERSTROKE study, but no results were presented for apoB levels.^3^ A recent study showed similar associations of LDL and apoB levels with ICH, and a Mendelian randomisation analysis indicated a causal relationship between LDL and ICH.^11^ It is not clear if the association between cholesterol and ICH differs for lobar and non-lobar ICH.^13,22^ In the present study, there was no clear difference for the effect of apoB between lobar and non-lobar ICH. It has been hypothesised that low levels of serum cholesterol causes fragile cerebrovascular endothelium.^24^ More studies are needed to elucidate the associations of different sub-fractions of plasma lipids and apolipoproteins with ICH, and with different locations of ICH.

Smoking was associated with all ICH and lobar ICH, but not non-lobar ICH, although a formal comparison of the effect of smoking on the two outcomes was non-significant. Zia et al. previously found that only lobar ICH was associated with current smoking.^9^ Few studies have found a significant association between smoking and ICH at all.^3,25–27^ However, most of them did not analyse lobar and non-lobar ICH separately. An association has been found between current smoking and incident cerebral microbleeds with lobar, but not non-lobar, location.^28^ It has been proposed that smoking causes ICH through formation of microaneurysms.^9^ It is unclear whether this only would pertain to lobar ICH. Hypothetically, an association of smoking with lobar vessel pathology may be related to cerebral amyloid angiopathy (CAA), which is an important cause of lobar ICH. It should be acknowledged that the effects of smoking with regard to obesity and blood pressure are complex. Many cross-sectional studies have reported lower weight and blood pressure in smokers, and smoking cessation has been associated with increased weight and blood pressure.^29,30^ These effects could also contribute to differences between studies on the association between smoking and ICH.

Diabetes was associated with a doubled risk of non-lobar ICH, even after multivariable adjustment, but not with all ICH. The association with diabetes was significantly different for lobar and non-lobar ICH. Some previous studies, including two meta-analyses, have reported non-significant associations between diabetes and risk of ICH.^5,31^ Two other meta-analyses have concluded that there may be an association between diabetes and ICH, but suggest that further information from large, population-based studies is required before the association can be confirmed.^32,33^ Another study found that high fasting glucose (>6.1 mmol/L or diagnosis of diabetes) was associated with incident non-lobar ICH, in accordance with our results.^34^ A recent Mendelian randomisation study suggested a relationship between diabetes and non-lobar ICH.^35^ Diabetes is associated with some imaging markers of cerebral small vessel disease (CSVD),^35^ and is also a risk factor for arteriolosclerosis.^36^ An association between diabetes and non-lobar ICH could hypothetically be explained by increased CSVD in the form of arteriolosclerosis, which is more prevalent in non-lobar than lobar ICH.^36^

As expected, use of oral anticoagulants was a strong risk factor for ICH in this study. In the subgroup analyses, only the association with lobar ICH was significant. However, use of oral anticoagulants at baseline was uncommon in this relatively young cohort. Only six cases of lobar ICH and two cases of non-lobar ICH used oral anticoagulants at baseline.

Being unmarried or divorced has been linked to cardiovascular diseases and mortality in many prospective studies, and many possible mechanisms have been proposed.^37^ Individuals who live alone might be less prone to seek medical help and less compliant to treatment. Unmarried individuals have worse health behaviour, with higher consumption of tobacco and alcohol.^38^ It has also been proposed that the social support offered by marriage could be protective *per se*. Living alone was significantly associated with ICH in this study, even after adjustment for e.g. smoking and alcohol consumption. The results suggest that living alone could increase the risk of ICH independently of other risk factors. It is also possible that living alone reflects a broad range of adverse health behaviours, which was not fully adjusted for in the analysis. Previous studies have linked psychosocial factors (stress, life events, depression) to increased risk of ICH.^3,39^ The reason behind the relationship between cohabiting status, psychosocial factors and risk of ICH warrants further investigation.

## Strengths and limitations

The cohort size and prospective design are important strengths of this study. The vast majority of cases were validated in medical records, including images, and subtyped based on ICH location. We used registers with national coverage, minimising the risk of loss to follow-up.

Similar to most cohort studies, participants in the MDCS tended to be healthier than non-participants. However, it is well known that this potential healthy selection bias is less important in prospective studies.^40^ As the MDCS cohort is predominantly Caucasian, it is uncertain if results can be generalised to other ethnic groups. Although many potential risk factors were adjusted for, residual confounding is still possible. Also, information about risk factors was only collected at one point in time, and some risk factors could have changed during the follow-up period. Many smokers quit smoking, and we can assume that many individuals with high blood pressure have received anti-hypertensive treatment during the follow-up. If anything, this should have biased the results towards null. Some of the ICH cases in this study may have been secondary due to causes that were not identified in the available workup. Furthermore, although this is a large prospective study with detailed information about the ICH cases, the possibility to draw certain conclusions about the differences in risk factors between lobar and non-lobar ICH is limited by the number of cases in these subgroups.

Lastly, while the classification of ICH as lobar or non-lobar is widely supported and used in many studies, it is also likely to be an oversimplification of the aetiology, adding further complexity to studies of ICH with different locations. The view that lobar ICH is caused primarily by CAA, and that non-lobar ICH is caused primarily by other CSVD related to hypertension, has been challenged.^41,42^ Non-lobar ICH tends to be caused by arteriolosclerosis, while lobar ICH is caused by both arteriolosclerosis and CAA.^43^

## Conclusion

In this prospective population-based study, high blood pressure, smoking, low apoB, use of oral anticoagulants and living alone were independently associated with incidence of spontaneous ICH. Diabetes, diastolic blood pressure and living alone were associated with an increased risk of non-lobar ICH only, while smoking was associated with lobar ICH. The associations with diabetes and diastolic blood pressure were significantly different between lobar and non-lobar ICH. Further research is required to elucidate how risk factors differ according to ICH location.

## Supplemental Material

sj-pdf-1-eso-10.1177_2396987320932069 - Supplemental material for Risk factors for intracerebral haemorrhage – Results from a prospective population-based study Click here for additional data file. Supplemental material, sj-pdf-1-eso-10.1177_2396987320932069 for Risk factors for intracerebral haemorrhage – Results from a prospective population-based study by Edith H Svensson, Kasim Abul-Kasim, Gunnar Engström and Martin Söderholm in European Stroke Journal
