# Supplementary material for: Risk factors for intracerebral haemorrhage – Results from a prospective population-based study
Source: Eur Stroke J. 2020 Jun 12;5(3):278–85. doi: 10.1177/2396987320932069 (PMC7538759; doi:10.1177/2396987320932069)
Supplement: sj-pdf-1-eso-10.1177_2396987320932069 - Supplemental material for Risk factors for intracerebral haemorrhage – Results from a prospective population-based study [file sj-pdf-1-eso-10.1177_2396987320932069.pdf]

Risk factors for intracerebral hemorrhage – results from a prospective population-based study

Edith H. Svensson, MD[1]
Kasim Abul-Kasim, MD, PhD[2,3]
Gunnar Engström, MD, PhD[1]
Martin Söderholm, MD, PhD[1,4]

1. Department of Clinical Sciences in Malmö, Lund University, Sweden
2. Radiology Diagnostics, Department of Translational Medicine, Malmö, Lund University, Sweden
3. Department of Radiology, Skåne University Hospital, Lund and Malmö, Sweden
4. Department of Neurology, Skåne University Hospital, Lund and Malmö, Sweden

Corresponding Author: Edith H. Svensson
edith.svensson@med.lu.se

Table 1. Hazard ratios for associations with lobar and non-lobar ICH

	Lobar ICH, age- and sex-adjusted HR (95 % CI)	Lobar ICH, multivariable adjusted* HR (95 % CI)	Non-lobar ICH, age- and sex-adjusted HR (95 % CI)	Non-lobar ICH, multivariable adjusted* HR (95 % CI)
Systolic BP, per 10 mmHg	1.11 (1.01–1.22)	1.13 (1.02–1.24)	1.23 (1.14–1.34)	1.22 (1.12–1.33)
Diastolic BP, per 10 mmHg	1.10 (0.91–1.33)	1.14 (0.94–1.39)	1.60 (1.37–1.87)	1.59 (1.35–1.88)
Hypertension	1.47 (0.94–2.28)	1.51 (0.97–2.37)	2.47 (1.60–3.83)	2.44 (1.57–3.80)
Diabetes mellitus	0.58 (0.19–1.85)	0.58 (0.18–1.84)	2.19 (1.24–3.88)	1.97 (1.10–3.53)
Current smoking	1.55 (1.04–2.31)	1.57 (1.04–2.36)	1.16 (0.80–1.67)	1.20 (0.82–1.75)
High alcohol intake	1.75 (0.81–3.79)	1.65 (0.76–3.60)	1.36 (0.66–2.79)	1.26 (0.61–2.61)
Educational level†				
Secondary	1.21 (0.76–1.94)	1.23 (0.77–1.96)	1.02 (0.66–1.57)	1.03 (0.66–1.61)
University	0.68 (0.34–1.35)	0.71 (0.35–1.42)	1.10 (0.68–1.78)	1.21 (0.74–1.98)
Living alone	0.93 (0.60–1.43)	0.89 (0.58–1.38)	1.44 (1.01–2.05)	1.43 (1.002–2.04)
BMI ‡				
<18.5 kg/m^2	1.56 (0.38–6.39)	1.53 (0.37–6.32)	–	–
25–30 kg/m^2	0.69 (0.46–1.04)	0.69 (0.46–1.05)	1.09 (0.77–1.56)	1.07 (0.75–1.53)
>30 kg/m^2	0.84 (0.48–1.46)	0.81 (0.46–1.43)	1.26 (0.78–2.02)	1.07 (0.66–1.75)
Waist circumference §	0.87 (0.51–1.49)	0.84 (0.48–1.45)	1.08 (0.69–1.70)	0.90 (0.57–1.44)
Low physical activity	1.36 (0.91–2.03)	1.38 (0.92–2.07)	0.96 (0.66–1.41)	1.10 (0.65–1.40)
Apolipoprotein A1 (per 10 mg/dL increase)	0.97 (0.90–1.04)	0.96 (0.89–1.04)	0.95 (0.89–1.02)	0.95 (0.89–1.02)
Apolipoprotein B (per 10 mg/dL increase)	0.95 (0.88–1.03)	0.94 (0.87–1.02)	0.99 (0.93–1.05)	0.97 (0.91–1.04)
Use of oral anticoagulants	7.36 (3.21–16.89)	8.18 (3.48–19.20)	1.89 (0.47–7.67)	1.92 (0.47–7.85)

ICH, intracerebral hemorrhage; HR, hazard ratio; CI, confidence interval; BP, blood pressure; BMI, body mass index; ApoB, apolipoprotein B; ApoA1, apolipoprotein A1.

* Main model adjusts for age, sex, systolic BP, use of BP-lowering drugs, current smoking, high alcohol intake, use of oral anticoagulants, diabetes mellitus, BMI and living alone. ApoA1 and ApoB also adjusted for lipid-lowering drugs. Diastolic BP not adjusted for systolic BP. Waist circumference not adjusted for BMI.

† Secondary (10–12 years) and university (>12 years) education compared to primary education (<=9 years).

‡ Compared to reference group (18.5–25 kg/m^2)

§ 4th compared to 1st sex-specific quartile.
